# Genetic variant in *microRNA-146a* gene is associated with risk of rheumatoid arthritis

**DOI:** 10.1080/07853890.2021.1933163

**Published:** 2021-06-01

**Authors:** Lin-Lin Zhang, Xiao-Xiao Wu, Xu-Fan Wang, Dong-Sheng Di, Qian Huang, Rui-Shan Liu, Zong-Wen Shuai, Dong-Qing Ye, Rui-Xue Leng

**Affiliations:** a Department of Epidemiology and Biostatistics, School of Public Health, Anhui Medical University, Hefei, PR China; b Inflammation and Immune Mediated Diseases Laboratory of Anhui Province, Hefei, PR China; c Department of Rheumatology and Immunology, the First Affiliated Hospital of Anhui Medical University, Hefei, PR China

**Keywords:** MiR-146a, single nucleotide polymorphisms, susceptibility, rheumatoid arthritis, cytokine

## Abstract

**Objective:**

To investigated the association between single nucleotide polymorphisms (SNPs) in *microRNA-146a* (*miR-146a*) gene and susceptibility of rheumatoid arthritis (RA).

**Methods:**

We systemically extracted the genetic data of *miR-146a* from previous genome-wide association studies (GWASs) of RA. Subsequently, we performed a replication study in an independent Chinese cohort for selected variant. A meta-analysis combined the previous GWASs with the replication study was also conducted. The epigenetic annotation and cytokine assay were used for exploring potential variant function.

**Results:**

The extracted genetic association data from three previous GWASs showed that the allele T of functional SNP rs2431697 increased RA susceptibility. The significant association for the SNP was also found in the Chinese replication cohort (OR = 1.24, 95% CI = 1.06–1.46, *p* = 8.69E-03). The estimated effect size for this SNP was larger in Asian population than that in European population (Asian meta-analysis: OR = 1.15, 95% CI = 1.09–1.22, *p* = 4.37E-07; Tran-ethnic meta-analysis: OR = 1.07, 95% CI = 1.04–1.10, *p* = 1.79E-06). The cytokine assay also showed that the risk allele T of the SNP rs2431697 is inversely associated with plasma TNF-α levels in health controls (*p* = .016).

**Conclusions:**

In summary, this study supports that genetic variant in *miR-146a* gene is associated with RA risk.

**KEY MESSAGES**
1. The association between SNPs in *miR-146a* gene and susceptibility of RA was unclear.
2. We investigated the genetic association using GWASs data and a replication study.
3. The SNP rs2431697 in *miR-146a* gene is associated with RA risk.

## 1. Introduction

Rheumatoid arthritis (RA) is a systemic, common autoimmune disease primarily characterized by synovial inflammation, autoantibody production and progressive bone destruction [1]. Although the exact causes of RA are still not completely understood, the genetic factors are known to be the major determinants of susceptibility to the disease [2].

MicroRNA-146a (miR-146a), a small non-coding RNA, is considered to be an important negative feedback regulator of innate immune responses and autoimmunity [3]. Recently, several genetic studies have investigated the relationship between single nucleotide polymorphisms (SNPs) in *miR-146a* gene and susceptibilities of several autoimmune diseases. Previous studies have suggested that the risk allele of rs2910164 is associated with increased risk of ankylosing spondylitis [4] and psoriasis [5]. Jazdzewski et al. [6] showed that the SNP rs2910164 G > C substitution in pre-miR-146a results in reduced amounts of mature miR-146a. The C allele also interferes with the binding of a nuclear factor to pre-miR-146a. Luo et al. [7] identified an association between a functional promoter variant (rs57095329) of *miR-146a* and risk of systemic lupus erythematosus (SLE) in Asian population. The risk-associated G allele had decreased binding to transcription factor Ets-1, contributing to reduced levels of *miR-146a* in SLE patients. Then, the genetic association of rs2431697 (in an intergenic region, between the *PTTG1* and *miR-146a*) with SLE was replicated in a case-control study in European population. Gene expression analysis revealed that the SNP rs2431697 is not associated with *PTTG1* expression levels, but with the *miRNA-146a*, where the risk allele correlates with lower expression levels of the miRNA [8]. Similar association for the SNP rs2431697 was also replicated in Chinese SLE population [9]. Moreover, Sun et al. [10] reported that the SNP rs2431697 is also associated with the risk of psoriasis in Chinese population. Hou et al. [11] found that the genomic region harbouring rs2431697 is a cell-type-specific enhancer specifically regulating miR-146a expression. The DNA sequence containing the rs2431697 C non-risk allele binds NF-κB with higher affinity and has greater accessibility relative to the rs2431697 T risk allele, thus driving increased expression of miR-146a. Besides, the study also suggests that the SLE risk mediated by the rs2431697-containing region may well act through *miR-146a-target* gene regulation within the type I interferon pathway.

Recently, systematic review and meta-analyses investigated the association of *miR-146a* gene polymorphisms with RA susceptibility. However, the review showed that only the SNP rs2910164 has been previously reported for RA susceptibility. Furthermore, no significant association for the SNP in the meta-analyses (*p* > .05) was found [12,13]. In contrast, available data showed that the expression levels of *miR-146a* are increased in peripheral blood mononuclear cells [14], synovial tissue [15], CD4+ T cells [16], IL-17 producing T cells [17] and RA synovial fibroblasts (RASFs) [18] in RA patients. Furthermore, functional studies also showed that administration of miR-146a prevents joint destruction in mice with collagen-induced arthritis (CIA) [19] and decreased expression of miR-146a contributes to an abnormal Treg phenotype in patients with RA [20]. Given that previous studies only investigated association of the SNP rs2910164 for susceptibility of RA and functional studies suggested that miR-146a contributes to aetiology of RA, we thus speculated that these previous studies might miss several genetic association signals in the loci. Herein, we will systemically estimate the association between variants of *miR-146a* gene and RA risk using the data of previous genome-wide association studies (GWASs) and a new replication study.

## 2. Materials and methods

### 2.1. Participants

The available data from three previous GWASs (European GWAS [GWAS_EUR: 14,361 RA cases and 43,923 controls], Japanese and Korean GWAS [GWAS_JP + KR: 4873 RA cases and 17,642 controls] and Chinese GWAS [GWAS_CN: 1027 RA cases and 2879 controls]) were used [21,22]. The summary statistics of GWAS_EUR and GWAS_JP + KR were available at the Japanese Encyclopaedia of Genetic associations by RIKEN (http://jenger.riken.jp/en/result). All the subjects in GWAS_CN were recruited from Anhui Province of China [22].

An independent replication cohort in Anhui (REP_AH: 779 cases and 1809 controls) was further recruited for selected SNP. During the study period (January 2014–June 2019), all questionnaires were obtained from the First Affiliated Hospital of Anhui Medical University or Anhui Provincial Hospital. The included RA patients were determined according to the revised criteria of the American College of Rheumatology 1987 criteria [23]. All patients included in this replication study have been diagnosed by two or more physicians in the Department of Rheumatology and Immunology of above hospitals. The exclusion criteria for RA patients are as follows: 1) patients with other autoimmune diseases; 2) patients with mental disease; 3) patients with chronic severe non-communicable diseases or systemic infections. The controls were people who did not suffer from RA and other autoimmune diseases in the same hospital during the same period. This study was conducted in accordance with the Declaration of Helsinki and approved by the medical ethics committee of Anhui Medical University. All subjects gave informed consent.

### 2.2. Genome-wide quality control and analyses

The details of genome-wide quality control and analyses for the three GWASs were available in previous studies [21,22]. For GWAS_CN, genotypes of autosomes were pre-phased using SHAPEIT2 and the imputation was performed using the IMPUTE version 2.0 software using 1000 Genomes October 2014 haplotypes (1000Genomes Phase 3) as the reference [24,25]. Association analysis of autosomes in GWAS_CN was performed by using SNPTEST with adjustment for age, sex and 10 principal components, by assuming an additive model [22,26]. The imputed SNPs were restricted based on minor allele frequency (MAF) > 0.005 and INFO scores > 0.7 [22]. The three GWASs were then meta-analysed with the inverse-variance method under a fixed-effect model with METAL [27].

### 2.3. Genotyping and analysis in an independent replication cohort

Selected SNP was genotyped in the independent cohort (REP_AH) using SNPscan technology (Genesky Biotechnologies Inc., Shanghai, China). Hardy–Weinberg equilibrium (HWE) test (*p* < .05) in controls will be conducted for quality control. We performed association analysis for REP_AH based on an additive model using PLINK version 1.07 (http://zzz.bwh.harvard.edu/plink/) [28]. The statistical power in the replication study will be calculated using Quanto version 1.2.4 [29].

### 2.4. Meta-analysis for the selected SNP

The three GWASs (GWAS_EUR, GWAS_JP + KR and GWAS_CN) and the replication cohort (REP_AH) were then meta-analysed with the inverse-variance method under a fixed-effect model using Stata version 14.2 (Stata Corp, College Station, TX). We conducted meta-analyses in Asian and trans-ethnic population, respectively. We described study heterogeneity using the *Q* and *I*^2^ statistic tests simultaneously.

### 2.5. Functional annotation and cytokine array

To further estimate potential regulatory roles of the likely causal variants in associated loci, we annotated lead SNP along with its surrounding correlated SNPs (*r*^2^ > 0.8), as implemented in HaploReg version 4.1 [30]. Plasma levels of four proinflammatory cytokines (TNF-α, IL-6, IL-17A and IFN-α) in 201 genotyped healthy controls were quantified using Human Luminex Screening Assay (R&D Systems, Minneapolis, MN). The association between expression levels of these cytokines and number of RA risk alleles was estimated by linear regression. Several common confounding factors (sex, age and test batches) were included as covariates in the regression model [22]. The statistical analysis was also performed using Stata version 14.2 (Stata Corp, College Station, TX).

## 3. Results

### 3.1. Genetic association of *miR-146a* gene with RA in GWASs

We firstly investigated genetic association of *miR-146a* gene region with RA risk using meta-analyses in Asian and trans-ethnic population, respectively. Of these identified top SNPs in trans-ethnic meta-analysis, only the association of SNP rs2431697 was significant in each GWAS (Table 1). The T allele of rs2431697 significantly increased the risk of RA. The pooled association of the SNP was also most significant in Asian meta-analysis (*p* = 9.77E-06). The association results of selected SNPs (rs57095329 and rs2910164) in each GWAS are also listed in Table 1. Both of them were associated with increased risk of RA in Europeans. However, the results were not significant in Asian population.

**Table 1.** Summary of statistical associations for the selected single nucleotide polymorphisms (SNPs) of *miR-146a* gene region in 3 GWASs.

SNP	CHR	Position (hg19)	A/B^a^	Study cohorts	OR (95% CI)	*p* Values
rs2431697	5	159,879,978	T/C	GWAS_EUR	1.04 (1.01–1.07)	7.82E-03
				GWAS_JP + KR	1.13 (1.06–1.21)	1.49E-04
				GWAS_CN	1.19 (1.03–1.37)	1.80E-02
rs57095329	5	159,894,847	G/A	GWAS_EUR	1.11 (1.01–1.23)	3.90E-02
				GWAS_JP + KR	1.05 (0.98–1.12)	1.62E-01
				GWAS_CN	1.04 (0.91–1.19)	5.53E-01
rs2910164	5	159,912,418	C/G	GWAS_EUR	1.06 (1.03–1.10)	5.55E-04
				GWAS_JP + KR	1.05 (0.99–1.10)	8.52E-02
				GWAS_CN^b^	1.01 (0.90–1.13)	8.71E-01

^a^Allele A is effect allele and allele B is reference allele.

^b^The SNP rs2961920 (*r*^2^ = 0.99) was selected as proxy SNP for the SNP rs2910164 in GWAS_CN.

CHR: chromosome; CN: Chinese; EUR: European; GWAS: genome-wide association study; JP: Japanese; KR: Korean; REP_AH: Anhui replication cohort; SNP: single nucleotide polymorphism.

### 3.2. Replication study for the SNP rs2431697

**Figure 1.**
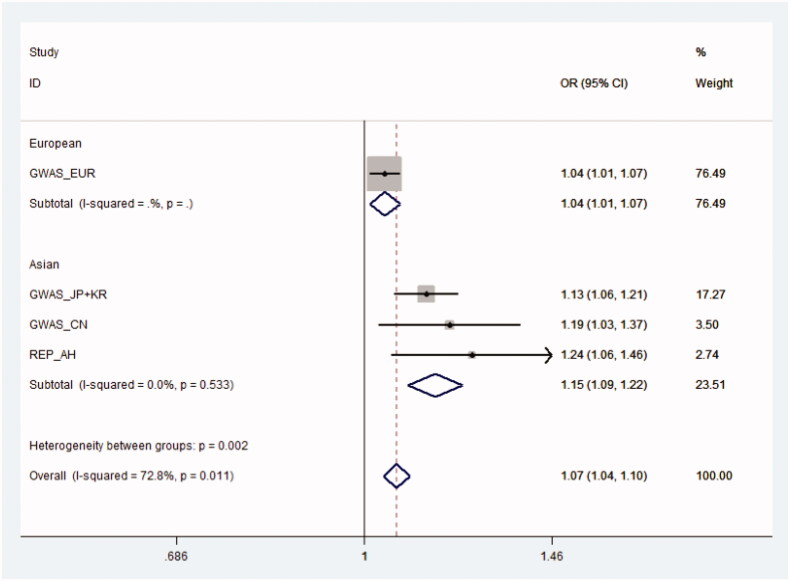
The association of the SNP rs2431697 with susceptibility of RA in meta-analyses. CN: Chinese; EUR: European; GWAS: genome-wide association study; JP: Japanese; KR: Korean; REP_AH: Anhui replication cohort.

To further validate the identified association, we selected the SNP rs2431697 in the replication study. The clinical and demographic features of the included subjects in the replication study are shown in Supplementary Table S1. As expected, significant association was found in REP_AH (OR = 1.24, 95% CI = 1.06–1.46, *p* = 8.69E-03). The *p* value for HWE test in controls was .082. Moreover, we performed a subgroup analysis of the relationship between SNP rs2431697 and RA in serologically positive patients in the replication cohort (a total of 580 RA patients and all controls). The effect size between rs2431697 and RA risk was still stable (OR = 1.25, 95% CI = 1.04–1.49, *p* = 1.67E-02). In this replication study (OR = 1.24, the frequency of risk allele = 0.83, the prevalence of RA in general population = 0.004), the statistical power will be 72.5%. Next, we performed an expanded meta-analysis with all results in the three GWASs and replication association study (REP_AH). The data synthesized in meta-analyses are presented in Figure 1. The pooled OR for rs2431697 was 1.15 (95% CI = 1.09–1.22; *p* = 4.37E-07) in Asian population and 1.07 (95% CI = 1.04–1.10, *p* = 1.79E-06) in trans-ethnic population. The effect size of the SNP rs2431697 was larger in Asian than that in European. No significant heterogeneity was observed across Asian population (*I*^2^ = 0.0%, *p* = .533). In contrast, a significant heterogeneity was found in the trans-ethnic meta-analysis (*I*^2^ = 72.8%, *p* = .011).

### 3.3. Functional annotation and cytokine assay

To further evaluate the regulatory role of the likely causal variants in the association loci, we annotated the lead SNP rs2431697 along with its surrounding correlated SNPs (*r*^2^ > 0.8), as implemented in HaploReg version 4.1. The data showed that the SNP rs2431697 was overlapped with multiple epigenetic marks in immune cells (Supplementary Table S2). It should be noted that the SNP is mainly overlapped with the enhancer marks in these cells. In addition, the cytokine assay also showed that the T risk allele of rs2431697 is inversely associated with plasma TNF-α levels in health controls (*p* = .016) (Table 2).

**Table 2.** Linear association between number of RA risk alleles and plasma levels of cytokines in 201 genotyped healthy controls.

RA risk SNPs	Test allele	Cytokines	*β*	*p* Values
rs2431697	T	TNF-α	−0.587	.016
		IL-6	−0.684	.397
		IL-17A	−0.077	.525
		IFN-α	−0.056	.234

SNP: single nucleotide polymorphism.

## 4. Discussion

In this study, we systemically estimated the association between SNPs in *miR-146a* gene and susceptibility of RA. We firstly identified that the T allele of the functional SNP rs2431697 increases RA risk. The effect direction of the SNP was consistent in the discovery and replication association study (Figure 1). Furthermore, we found that the effect size of the lead SNP is larger in Asians than that in Europeans. Consistently, significant heterogeneity was found in trans-ethnic meta-analysis (*I*^2^ = 72.8%, *p* = .011) but not in Asian meta-analysis (*I*^2^ = 0.0%, *p* = .533).

Of note, the cytokine assay in this study also showed that the T risk allele of rs2431697 is inversely associated with plasma TNF-α levels in healthy controls. We judged that the inverse correlation may not derive from common confounding bias since the possible factors (sex, age and test batches) were included as covariates in the regression model. Although previously functional study showed that miR-146a overexpression can significantly reduce TNF-α levels in the supernatant of the cell culture medium of THP-1 cells following LPS treatment [31]. However, the findings of the cytokine assay in this study suggested that miR-146a levels are positively associated with TNF-α production in plasma since the T allele of rs2431697 is associated with decreased expression of *miR-146a* [8]. Consistently, Li et al. [16] measured the levels of IL-2, IL-4, IL-6, IL-10, IFN-γ and TNF-α in both synovial fluid (SF) and serum of RA patients and healthy donors. They found that only *miR-146a* expression in CD4+ T cells is positively correlated with the levels of TNF-α in both SF and peripheral blood of RA patients, but not associated with the levels of other cytokines. We further speculated that the inverse association might be attributable to reverse causality since *in vitro* studies showed that TNF-α stimulation (a known stimulus of NF-kB signalling in these cells) upregulated *miR-146a* expression in Jurkat T cells and human CD4+ T cells in a dose-dependent fashion [16]. The similar finding was also observed in other studies [15,32].

The following advantages of this study ensured the credibility of the results. First, by using large-scale GWASs data, we can systemically estimate the association between SNPs in *miR-146a* gene and susceptibility of RA in different populations. Second, the initial genetic findings were replicated in an independent genotyped data, which strengthened the validation of the conclusion. Meanwhile, there are, however, also several limitations. First, the unavailable individual data in previous GWAS studies may limit conditional association analyses in meta-analysis level [21,22]. Second, extra-articular manifestations, joint erosions, DAS28 and other indicators of disease activity were not collected in our replication study.

In summary, our data firstly supported that the SNP rs2431697 in *miR-146a* gene correlates with susceptibility of RA. The detailed mechanism of the loci in the pathogenesis of RA needs to be further studied.

## Supplementary Material

Supplemental Material

## Data Availability

The data of Chinese population that support the findings of this study are available from the corresponding authors, upon reasonable request.
