# Supplementary material for: Genetic variant in microRNA-146a gene is associated with risk of rheumatoid arthritis
Source: Ann Med. 2021 Jun 1;53(1):824–9. doi: 10.1080/07853890.2021.1933163 (PMC8172213; doi:10.1080/07853890.2021.1933163)
Supplement: Supplemental Material [file IANN_A_1933163_SM7612.doc]

**Supplementary Table S1**. Characteristics of the subjects included in the Anhui replication cohort.

| Characteristics | Case (n=779) ^†^ | Control (n=1809) ^†^ |
| --- | --- | --- |
| Age, yrs | 52.96 ± 13.34 | 40.74 ± 11.40 |
| Sex |  |  |
| Female | 386(83.55) | 1508(83.36) |
| Male | 76(16.45) | 301(16.64) |
| Diagnose age, yrs | 45.80 ± 14.18 | - |
| Duration, yrs | 7.57 ± 8.09 | - |
| RF |  |  |
| + | 529(86.00) | - |
| - | 86(14.00) | - |
| ACPA |  |  |
| + | 495(84.90) | - |
| - | 88(15.10) | - |

RF, rheumatoid factors; ACPA, anti-cyclic citrullinated peptide antibody.

^†^Values are given as mean ± SD or number (percentage).

**Supplementary Table S2**. The overlap of the new loci with epigenetic marks in immune cells using HaploReg annotation.

| **Immune cell types** | **Roadmap core 15-state model**  **(Enhancers)** | **Enhancer**  **mark**  **H3K4me1** | **Enhancer**  **mark**  **H3K27ac** | **Promoter**  **mark**  **H3K4me3** | **Promoter**  **mark**  **H3K9ac** | **DNase**  **I-hypersensitive**  **sites** |
| --- | --- | --- | --- | --- | --- | --- |
| Primary T cells from peripheral blood |  |  |  |  |  |  |
| Primary T regulatory cells from peripheral blood |  |  |  |  |  |  |
| Primary monocytes from peripheral blood |  |  |  |  |  |  |
| Primary B cells from peripheral blood |  |  |  |  |  |  |
| GM12878 Lymphoblastoid Cells |  |  |  |  |  |  |
| Monocytes-CD14+ RO01746 Primary Cells |  |  |  |  |  |  |
